# The *SlFSR* gene controls fruit shelf-life in tomato

**DOI:** 10.1093/jxb/ery116

**Published:** 2018-04-04

**Authors:** Lincheng Zhang, Mingku Zhu, Lijun Ren, Anzhou Li, Guoping Chen, Zongli Hu

**Affiliations:** Key Laboratory of Biorheological Science and Technology (Chongqing University), Ministry of Education, Bioengineering College, Chongqing University, Chongqing, China

**Keywords:** Cell wall metabolism, GRAS transcription factor, overexpression, RNAi, shelf-life, *SlFSR*, tomato

## Abstract

Fruit ripening represents a process that changes flavor and appearance and also a process that dramatically increases fruit softening. Fruit softening and textural variations mainly result from disruptions to the cell walls of the fruit throughout ripening, but the exact mechanisms and specific modifications of the cell wall remain unclear. Plant-specific GRAS proteins play a critical role in development and growth. To date, few GRAS genes have been functionally categorized in tomato. The expression of a novel GRAS gene described in this study and designated as *SlFSR* (*fruit shelf-life regulator*) specifically increased during fruit ripening, but was significantly decreased in the tomato mutant *rin* (*ripening inhibitor*). RNAi repression of *SlFSR* resulted in reduced expression of multiple cell wall modification-related genes, decreased the activities of PG (polygalacturonase), TBG (tomato β-galactosidase), CEL (cellulase), and XYL (β-D-xylosidase), and significantly prolonged fruit shelf-life. Furthermore, overexpression of *SlFSR* in mutant *rin* gave rise to up-regulated expression of multiple cell wall modification-related genes, such as *PG*, *TBG4*, *CEL2*, *XYL1*, *PL*, *PE*, *MAN1*, *EXP1*, and *XTH5*, and significantly shortened the fruit shelf-life. These findings reveal some of the genetic mechanisms underlying fruit cell wall metabolism and suggest that the *SlFSR* gene is another potential biotechnological target for the control of tomato fruit shelf-life.

## Teaser

A tomato GRAS transcription factor, designated as *SlFSR* (*fruit shelf-life regulator*), controls fruit shelf-life by regulating the expression of cell wall modification-related genes and metabolism of pectin and cellulose.

## Introduction

Fruits contain essential key nutrients of the diet of humans and many animals. Fruit ripening is a stepwise growth process that involves complex changes in physiological and metabolic processes such as fruit softening, carotenoid accumulation, chlorophyll degradation, and flavor biosynthesis. All these changes lead to the fruit developing the required quality for consumption, but the shelf-life of fruit is determined by how long these required features last. The main cause of fruit rotting is the extent of softening. The cost of fruit is also dependent on the extent of softening because it has a direct effect on palatability, shelf-life, resistance to post-harvest pathogen infection, transportation, storage, and consumer acceptability (Brummell and Harpster, 2001; Meli *et al.*, 2010). Tomato belongs to the group of soft fruits characterized by a rapid and high loss of firm texture during the ripening process but is one of the most commonly used and versatile fruits in terms of its nutritional and commercial value. In addition, tomato has long served as an excellent model of fruit ripening and softening in research, primarily due to its small genome, efficient transient and stable transformation, short life cycle, well-characterized ripening mutants, rich genomic resources, and commercial importance (Moore *et al.*, 2002; Giovannoni, 2007; Sato *et al.*, 2012; Zhu *et al.*, 2014).

Physiologically, tomato is a typical climacteric fruit and its ripening is determined by proper softening, which is caused by short-term ethylene biosynthesis and higher respiration (Adams-Phillips *et al.*, 2004). Studies that have identified various mechanisms that control fruit ripening have greatly benefited from the availability of numerous ripening-deficient mutants, which have been very valuable in exploring the roles of cell wall-modifying proteins and changes in cell wall during softening. These mutants include *rin* (Vrebalov *et al.*, 2002), *Nr* (*never ripe*) (Wilkinson *et al.*, 1995), *nor* (*non-ripening*) (Giovannoni, 2007), and *Cnr* (*Colorless non-ripening*) (Orfila *et al.*, 2001).

Fruits of *rin* and *nor* mutants do not show increases in lycopene and carotenes, and soften very gradually. Studies of ripening control mechanisms have shown that the *RIN* and *NOR* genes both act upstream of ethylene, and regulate both ethylene and non-ethylene-controlled functions (Tigchelaar *et al.*, 1978; Moore *et al.*, 2002). *RIN* encodes a MADS-box transcription factor, which is considered as a pivotal regulator of tomato fruit ripening (Vrebalov *et al.*, 2002). However, both *rin* and *nor* fruits are unpalatable and have poor quality, which limits their commercial value. Therefore, genetic studies involving the transfer of genes to regulate specific genes involved in fruit softening are one of the major areas of biological research. This approach can also potentially reduce the level of fruit softening while permitting the accumulation of the necessary components of normal ripening (i.e. sugars, pigments, volatiles, and organic acids), increase shelf-life, and decrease spoilage rate. Recently, the suppression of cell wall modiﬁcation-related genes has been used to reduce the softening of fruit in transgenic tomato (Han *et al.*, 2016; Uluisik *et al.*, 2016; Yang *et al.*, 2017). However, these studies have had very little success. Disruption of the cell wall is mainly responsible for fruit softening and textural variations throughout ripening, but the exact mechanisms and particular functions of cell wall modifications during fruit ripening are still poorly understood. In addition, the improvements to fruit shelf-life accomplished to date have not been sufficient, thus the identiﬁcation of more targets is required.

The GRAS proteins are a recently identified plant-specific family of putative transcription factors, whose name derives from the three initially identiﬁed members, GAI (gibberellic acid insensitive), RGA (repressor of GAI), and SCR (scarecrow) (Pysh *et al.*, 1999). Typically, GRAS proteins are composed of 400–770 amino acid residues, and the GRAS domain contains five conserved motifs, including LHRI, VHIID, LHRII, PFYRE, and SAW (Bolle, 2004). To date, genes encoding GRAS proteins have been studied primarily in the model plants Arabidopsis and rice, in which 34 and 60 putative GRAS members have been identiﬁed, respectively (Liu and Widmer, 2014). Plant molecular genetics studies have shown that GRAS genes play various critical roles in growth and development, such as in root development, phytohormones, light signaling pathways, and transcriptional regulation in response to biotic and abiotic stress (Bolle, 2004; Smit *et al.*, 2005; Ma *et al.*, 2010; Sun *et al.*, 2012; Liu and Widmer, 2014); for example, *AtSCL3* (a member of the AtSCL3 subfamily) in assimilating several signals in root cell elongation of Arabidopsis (Heo *et al.*, 2011), and *DLT* (a member of the DLT subfamily) in brassinosteroid signaling of rice (Tong *et al.*, 2009). In addition, it has been reported that the overexpression of the *Populus euphratica* gene *PeSCL7* (AtSCL4/7 subfamily) in transgenic Arabidopsis boosts drought and salt tolerance (Ma *et al.*, 2010). Recently, a GRAS protein known as RAM1 has been considered essential for infection by arbuscular mycorrhizal fungi (Gobbato *et al.*, 2012). Although the GRAS proteins are encoded by a large gene family and have been studied for several years, currently we have only an incomplete understanding of many of their features, and the specific biological functions of most members remain unclear.

Few GRAS family members have been functionally categorized in tomato, and the contribution of GRAS proteins to fruit ripening and/or softening has not been reported to date. Previously, 17 putative tomato GRAS genes were identified by Mayrose *et al.* (2006). *Pseudomonas syringae* pv. *tomato* was used to up-regulate six *SlGRAS* transcripts and the transcripts of eight *SlGRAS* genes increased in response to mechanical stress. Suppression of *SlGRAS6* impaired tomato resistance to *P. syringae* pv. *tomato*. The first characterized tomato GRAS gene, *Ls* (*Lateral suppressor*), is obligatory for the initiation of axillary meristems (Mayrose *et al.*, 2006). A gibberellin (GA)-constitutive-response tomato mutant *pro* (*procera*) carries a point mutation in the GRAS region of the gene encoding SlDELLA, a repressor in the GA signaling pathway, which was shown to function in the control of flower morphology, cell division, expansion, and the auxin-signaling pathway throughout fruit set and growth (Brummell and Harpster, 2001; Carrera *et al.*, 2012). *Solyc07g052960* was reported by Fei *et al.* (2004) to be the direct target of RIN, and it was revealed to be a ripening-specific GRAS gene. More recently, *Solyc07g052960* was also identified as a direct target of RIN and subjected to qChip–PCR, which showed a high degree of RIN dependence, the highest FCWT value (683.5), and the highest ECS value (100.6) among the gene category associated with transcription factors (Fujisawa *et al.*, 2013; Fujisawa *et al.*, 2012). In this study, we explored the function of this gene, named as *SlFSR* (*fruit shelf-life regulator*), which was isolated from tomato fruit by a cDNA clone, and whose mRNA specifically accumulates in ripening fruits. RNAi repression of *SlFSR* was accomplished to further examine its role in tomato. In *SlFSR*-RNAi fruits, decreased expression of multiple cell wall modification-related genes, reduced PG (polygalacturonase), TBG (tomato β-galactosidase), CEL (cellulase), and XYL (β-D-xylosidase) activities, and significantly enhanced shelf-life were detected. A *SlFSR*-overexpressing *rin* mutant was also generated, in which the overexpression of *SlFSR* resulted in the up-regulation of multiple cell wall modification-related genes, including *PG*, *TBG4*, *CEL2*, *XYL1*, *pectate lyase* (*PL*), *pectinesterase* (*PE*), *mannosidase* (*MAN1*), *xyloglucan endotransglucosylase/hydrolase* (*XTH5*), and *expansin 1* (*EXP1*), and significantly shortened fruit shelf-life. These findings suggest that *SlFSR* plays an essential role in fruit post-harvest storage, and its underlying molecular mechanisms involved in fruit cell wall modification are discussed. Our results also indicate another possible biotechnological approach to extend fruit shelf-life, in addition to altering ethylene biosynthesis and cell wall metabolism.

## Materials and methods

### Promoter analysis of *SlFSR* in tomato

To study the putative *cis*-elements in the promoter region of the *SlFSR* gene, the promoter sequence (2 kb region upstream of the 5ʹ end of the predicted open reading frame) of *SlFSR* was extracted from the SGN catalog (https://solgenomics.net/; accessed 20 October 2017) and searched against the promoter database PLACE (http://www.dna.affrc.go.jp/PLACE/index.html; accessed 20 October 2017) (Higo *et al.*, 1999).

### Plant materials and growth conditions

The wild-type (WT) tomato *Solanum lycopersicum* Mill. cv. Ailsa Craig, *rin* and *Nr* mutants, *SlFSR*-RNAi, and *SlFSR*-overexpressing transgenic lines were grown in a greenhouse under the following conditions: 16 h day (27 °C) and 8 h night (19 °C), at 80% relative humidity; plants were irrigated regularly. For tissue-specific expression of *SlFSR*, leaves, flowers, sepals, roots, and fruits at various stages of development were gathered. Flowers were sampled at anthesis. Fruit development was denoted as days post anthesis (DPA). Fruits at 20 DPA were defined as immature green (IMG). Fruits at 35 DPA were defined as mature green (MG) and considered as full fruit growth but with no clear ripe fruit color evident. Breaker (B) fruit was recorded as fruit with the first appearance of orange color. The following ripening periods were distinguished as B+4 (4 days after breaker) and B+7 (7 days after breaker). WT and *rin* lines were used to produce *SlFSR*-RNAi and *SlFSR*-overexpressing transgenic lines, respectively. Fruits from the *Nr* and *rin* mutants were harvested at IMG, MG, B, B+4, and B+7 stages when they showed equivalent characteristics to those defined in WT tomato. All samples were immediately transferred to liquid nitrogen and stored at –80 °C until required.

### Construction of RNAi and overexpression vectors and plant transformation

The *SlFSR* RNAi and overexpression constructs were made using the pBIN19 and pBI121 vectors, respectively, as described previously (Dong *et al.*, 2013; Xie *et al.*, 2014). The detailed method was as follows: for the RNAi vector construction, a 718 bp fragment of DNA was amplified with *SlFSR*-RNAi-F/R primers (see Supplementary Table S1 at *JXB* online) which had been joined with *Kpn*I/*Cla*I and *Xho*I/*Xba*I restriction sites at the 5ʹ end. The amplified products were digested with the restriction enzymes *Cla*I/*Xba*I and *Kpn*I/*Xho*I and linked to the plasmid pHANNIBAL using the same restriction enzymes. The double-stranded RNAi expression unit was digested with the restriction enzymes *Sac*I/*Spe*I and inserted into the plant binary vector PBIN19 via *Sac*I/*Xba*I restriction sites to form the RNAi vector. For construction of the overexpression construction, the full-length cDNA of *SlFSR* was amplified with *SlFSR*-over-F/R primers to which *Xba*I/*Sac*I restriction sites were inserted at the 5ʹ end (Supplementary Table S1). The amplified products were digested with *Xba*I/*Sac*I and linked to the plant binary vector pBI121 at *Xba*I/*Sac*I restriction sites. Finally, the RNAi vector was transformed into WT tomato and the overexpression vector was transformed into the tomato mutant *rin* through the freeze-thaw method, using *Agrobacterium tumefaciens* strain LBA4404 (An, 1987). Transgenic lines were selected on the basis of kanamycin (50 mg l^−1^) resistance. Genomic DNA of the WT and transgenic lines was isolated using a kit (Invitrogen, Shanghai, China) and the presence of T-DNA was confirmed by PCR using *NPTII*-F/R primers (Supplementary Table S1).

### Total RNA extraction and qRT–PCR analysis

Total RNA was extracted from various samples using Trizol reagent (Invitrogen, Shanghai, China). First-strand cDNA was synthesized using a kit (Promega, Beijing, China). Quantitative reverse-transcription–PCR (qRT–PCR) was performed by using a CFX96™ Real-Time System (Bio-Rad, USA). The reaction mixture consisted of 5 μl enzyme solution (2×GoTaq^®^ qPCR Master Mix, Promega, Beijing, China), 1 μl cDNA, 0.5 μl primer pairs (10 mM), and 3.5 μl distilled water. The reaction conditions were 95 °C for 3 min, followed by 40 cycles of 95 °C for 15 s and *T*_m_ (the most suitable temperature for each gene) for 45 s, followed by a melting curve analysis. The *CAC* gene of tomato was used as an internal control for expression analysis (Expósito-Rodríguez *et al.*, 2008; Nicot *et al.*, 2005), and the 2^–ΔΔCT^ method was used for the analysis of relative expression levels (Livak and Schmittgen, 2001). A no-template control was also included in each gene study. All qRT–PCRs were performed in three replicates. The primers used for each gene are listed in Supplementary Table S1; a standard curve was performed for each pair of specific primers.

### Enzyme determination assays

For all enzyme determination assays, 0.1 g of fresh pericarp at the B+4 stage was ground in an ice water bath. The activity of PG, TBG, CEL, and XYL in *rin*, *SlFSR* transgenic lines, and WT tomato was analyzed using a kit (Komin Suzhou, China) according to the manufacturer’s instructions. Three individual fruits were sampled from each line and the assays were done in triplicate.

### Metabolite analysis

For analysis of the quantity of pectin, 3 mg of pericarp at the B+4 stage was ground in liquid nitrogen. Total pectin, water-soluble pectin, cellulose, and hemicelluose were analyzed using a kit (Komin Suzhou, China) according to the manufacturer’s instructions. The levels of soluble sugar in the fruit were determined exactly as described previously (Fernie *et al.*, 2001). Malic acid and citric acid contents were measured as described by Nunes-Nesi *et al.* (2007). Three independent fruits at the B+4 stage were sampled and the assays were performed in triplicate.

### Ethylene measurement

The *rin* mutant, WT, and transgenic lines were harvested at the B, B+4, and B+7 stages and kept at room temperature for 3 h to reduce the influence of wound-induced ethylene produced in response to harvesting of the fruits. The fruits were weighed and then placed in 235 ml glass jars sealed with a plastic membrane and stored for 24 h at room temperature(Zhu *et al.*, 2014). The ethylene concentration in a 1 ml sample of headspace gas from each glass jar was measured by using the method of Chung *et al.* (2010).

### Pigment extraction

Carotenoids were extracted from a 5 mm wide rectangular strip of freeze-dried pericarp, sampled from around the equator of fruits, according to an improved protocol described by Forth and Pyke (2006). Each sample (of known weight) was ground into a powder in liquid nitrogen and then placed into a 2 ml tube. Pigments were extracted by the addition of hexane:acetone (6:4, v/v). The sample was then centrifuged at 2500 *g* for 5 min and the supernatant was placed in a new tube after centrifugation. The sediment was repeatedly extracted with hexane:acetone (6: 4, v/v) until it was colorless. The absorbance of the supernatant was immediately measured. The total carotenoids content was quantified using the equation: total carotenoids (mg ml^–1^)=4×(OD_450_)×10 ml/1 g. All experiments were repeated for individual samples at least three times.

### Water loss measurements

Nine fruits from WT tomato and each of the *SlFSR*-RNAi lines were collected at the B+4 stage, and nine fruits from *rin* and each of the overexpressing lines were harvested at the B stage. Fruits of WT and *SlFSR*-RNAi lines were kept at room temperature (23–25 °C with 55–60% relative humidity) for 2 months after harvest; fruits of *rin* and *SlFSR*-overexpressing lines were stored at room temperature for 3 months after harvest. Water loss per unit fruit weight was calculated after recording the weight decrease over time. The weight loss of WT and *SlFSR*-RNAi fruits was measured at 0, 7, 10, 13, 16,19, 22, 25, 28, 31, 34, 37, and 40 days.

### Storage assays of tomato fruits

Fruits of WT and *SlFSR*-RNAi lines were harvested at the B+4 stage, and fruits of *rin* and *SlFSR*-overexpressing lines were harvested at the B stage. All the fruits were disinfected with 10% bleach for 10 min, followed by rinsing with sterilized water and air-drying. The fruits of WT and *SlFSR*-RNAi lines were stored at room temperature for 2 months; fruits of *rin* and *SlFSR*-overexpressing lines were stored at room temperature for 3 months. The chromatic softening and collapse of the fruits were evaluated by taking photographs at the beginning (day 7 after harvesting) and end (day 90 after harvesting) of the storage period.

### Microscopic observations

Approximately 2 cm of pericarp was collected from fruits stores for 2 months (WT and RNAi lines) or 3 months (*rin* and overexpressing lines). Samples were immediately ﬁxed in FAA liquid (70% ethanol, acetic acid, and formaldehyde mixed 18:1:1 v/v) and subsequently dehydrated, wax embedded, sectioned, dewaxed, and stained with safranin and fast green. All observations were made under a light microscope (Olympus IX71, Japan) and photographed. Three replicates were performed for each sample.

### Statistical analysis

Data were subjected to analysis of variance with SPSS Statistics 18.0. Differential expression levels were considered to be statistically significant when exceeding the Dunnett’s test critical value at the *P*<0.05 level. The difference was defined as ‘repressed’, ‘induced’, or ‘different’ only if such differences met the above standard.

## Results

### Expression profiles of *SlFSR* in WT tomato

**Fig. 1.**
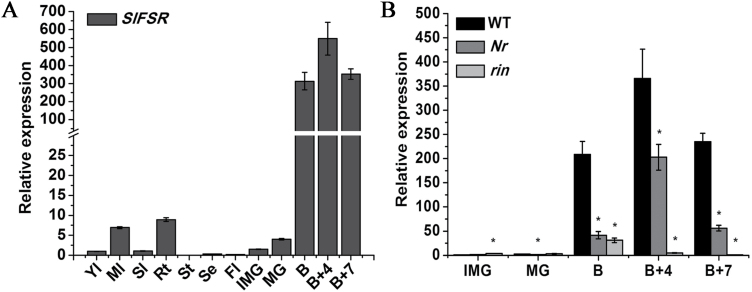
(A) Expression of *SlFSR* in different tissues of WT tomato. (B) Expression of *SlFSR* in WT and ripening mutant fruits. Total RNA from *rin* and *Nr* fruits at the IMG, MG, B, B+4, and B+7 stages equivalent to WT tomato was subjected to qRT–PCR analysis. B, Breaker stage; B+4, 4 days after breaker stage; B+7, 7 days after breaker stage; Fl, flower; IMG, immature green; MG, mature green; Ml, mature leaf; Rt, root; Se, sepal; Sl, senescent leaf; St, stem; Yl, young leaf. Data are the mean ±SE of three independent experiments. Significant differences (*P*<0.05) are denoted by asterisks.

The expression proﬁles of the *SlFSR* gene in different tissues of WT tomato were detected by qRT–PCR. *SlFSR* mRNA was predominantly expressed in the fruit during the ripening stages (B, B+4, and B+7), but little or no expression was observed in all other tissues (Fig. 1A). The B stage sees the first ripening-related changes in tomato fruit due to climacteric changes in ethylene production, cell wall disruption, and synthesis of lycopene, followed by an obvious increase in the expression level of cell wall hydrolases (Fischer and Bennett, 1991; Giovannoni, 2004). Consequently, higher expression of *SlFSR* at the ripening stage indicates its role in tomato fruit ripening and softening.

### Expression of *SlFSR* is inhibited in tomato ripening mutants and regulated by ethylene

The level of *SlFSR* expression increased mainly in the ripening stage of tomato fruit; this led us to examine its expression in the ripening-impaired mutants *rin* (in which higher ethylene is not produced and ripening activities are affected) and *Nr* (which is unresponsive to ethylene). As in WT fruits, almost undetectable *SlFSR* gene transcription was observed in IMG and MG fruits of *rin* and *Nr* mutants (Fig. 1B). *SlFSR* expression was significantly down-regulated in both *rin* and *Nr* (especially in *rin*) (Fig. 1B), indicating that *SlFSR* expression is obstructed by both the *RIN* and *Nr* mutations. The reduced expression of *SlFSR* strongly indicates its contribution to fruit ripening and induction by ethylene. Indeed, a putative ethylene responsive element was found in the promoter sequence of *SlFSR* (Supplementary Fig S1). These observations indicate the relationship between ethylene and *SlFSR* expression, and the action of ripening regulators downstream of *SlFSR*.

### *SlFSR*-RNAi fruits go through normal climacteric ripening and color development

**Fig. 2.**
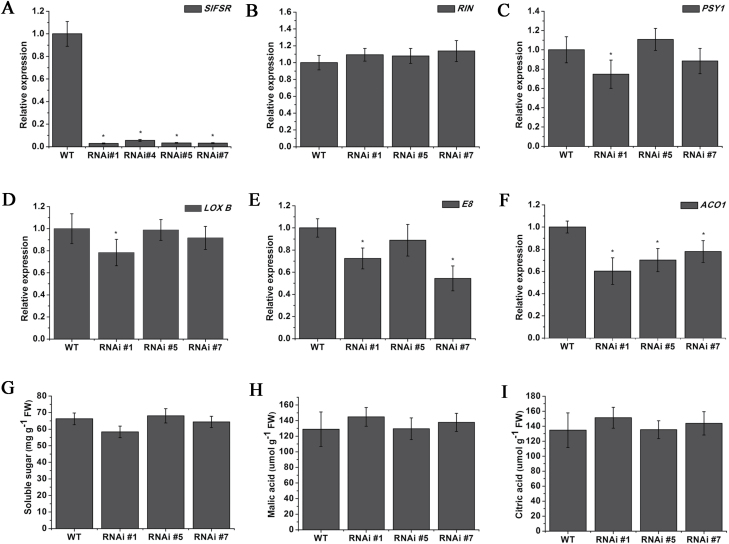
Silencing of *SlFSR* in WT tomato causes no obvious phenotypic changes. Relative expression profiles of *SlFSR* (A) and ethylene- and ripening-related genes (B–F) in WT and *SlFSR*-RNAi fruits at B+4 stage. (G–I) Analysis of flavor compound contents in WT and *SlFSR*-RNAi fruits at B+4 stage. The expression data for WT plants were normalized to a value of 1. Each value represents the mean ±SE of three replicates. Asterisks indicate significant differences (*P*<0.05) between WT and RNAi lines.

To further study the role of the *SlFSR* gene, several independent RNAi silencing lines were obtained. The accumulation of *SlFSR* transcript was greatly silenced, to approximately 2–5% of control levels at the B+4 stage, in the RNAi lines (Fig. 2A). Curiously, regardless of the specific accumulation of *SlFSR* in ripening fruits, the significantly silencing of *SlFSR* had no apparent effect on the tomato fruit ripening phenotype (data not shown). It is known that the production, perception, and transfer of ethylene signals are required for complete fruit ripening (Alexander and Grierson, 2002), and thus the expression levels of ripening-related genes were assessed in the *SlFSR*-RNAi lines. The expression of *PHYTOENE SYNTHETASE1* (*PSY1*) (Fray and Grierson, 1993), *RIN* (Vrebalov *et al.*, 2002), and *TomloxB* (Griffiths *et al.*, 1999) were almost unchanged in *SlFSR*-RNAi lines relative to expression in WT tomato, but expression of *1-AMINOCYCLOPROPANE-1-CARBOXYLATE OXIDASE* (*ACO1*) (Barry and Giovannoni, 2007) and *E8* (Lincoln *et al.*, 1987) was significantly reduced in *SlFSR*-RNAi fruits (Fig. 2B–F). This slight reduction apparently did not affect the phenotype of *SlFSR* fruits. Moreover, some compounds characteristic of flavor, such as sugar, malic acid, and citric acid were measured in *SlFSR*-RNAi fruit, and no significant difference was found in the contents of each of them relative to WT fruit (Fig. 2G–I).

### Silencing of *SlFSR* greatly extends tomato fruits shelf-life

**Fig. 3.**
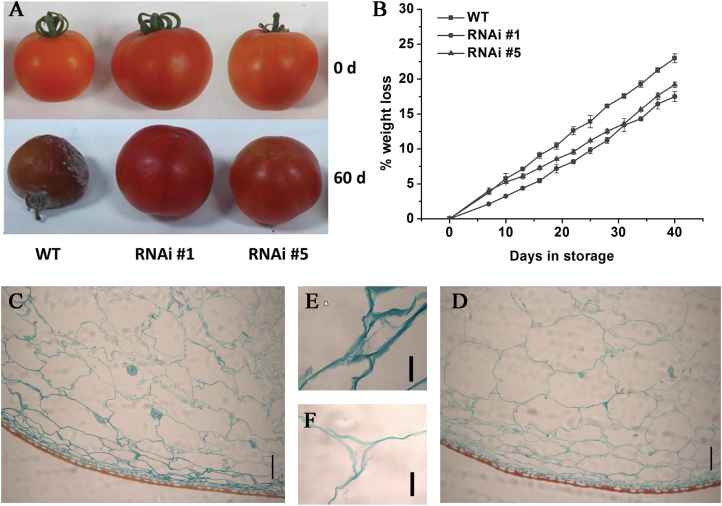
Silencing *SlFSR* alters cell wall components and increases shelf-life of tomato fruit. (A) WT and *SlFSR*-RNAi fruits harvested at B+7 stage were stored at room temperature for 60 days. (B) Weight loss of WT and *SlFSR*-RNAi fruits during storage. (C, E) Microscopic observations of WT tomato fruit stored for 40 days. (D, F) Microscopic observations of *SlFSR*-RNAi tomato fruit stored for 40 days. (C, D) Bar=50 μm; (E, F) Bar=25 μm.

To assess the shelf-life of *SlFSR*-RNAi fruits, storage tests were performed using B+4 fruits at room temperature. After 2 months of storage, WT tomato fruits were completely collapsed and severely infected, while *SlFSR*-RNAi fruits showed delayed signs of deterioration and no visible infection was observed (Fig. 3A). In addition, the RNAi lines showed obviously lower weight loss (Fig. 3B). Microscopic examination of pericarp revealed ruptured cells with an irregular shape in WT fruits after 40 days of storage (Fig. 3C), while the cells of the RNAi lines were comparatively round shaped and normal in appearance (Fig. 3D). In addition, the cell wall of WT fruits was degraded, while at this point the *SlFSR*-RNAi fruits showed a normal cell wall structure (Fig. 3E, F). These results indicate that silencing *SlFSR* in tomato is sufficient to change the post-harvest ripening process and greatly prolong fruit shelf-life.

### Expression profiles of cell wall modiﬁcation-related genes in *SlFSR*-RNAi fruits

**Fig. 4.**
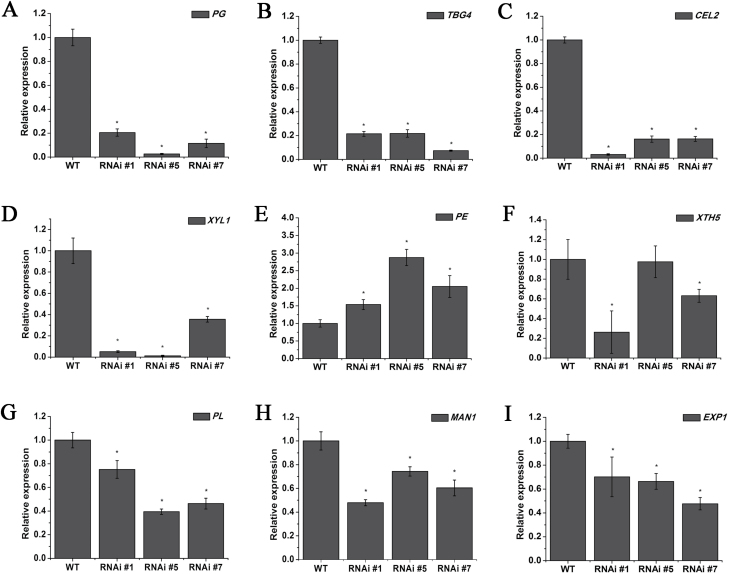
(A–I) Relative expression profiles of cell wall metabolism genes in the pericarp of WT and *SlFSR*-RNAi tomato fruits. The expression data for WT plants were normalized to a value of 1. Each value represents the mean ±SE of three replicates. Asterisks indicate significant differences (*P*<0.05) between WT and RNAi lines.

Fruit ripening is associated with cell wall modifications (Orfila *et al.*, 2002). Tomato cell wall modiﬁcation involves processes including depolymerization and solubilization of pectins and hemicellulosic polysaccharides (Brummell, 2006). This process is stimulated by various cell wall modifying enzymes and proteins, including PG, CEL, TBG, XYL, XTH, and PE, and other cell wall loosening proteins, such as EXP (Brummell and Harpster, 2001). To investigate whether the expression of cell wall modiﬁcation-related genes differs between *SlFSR*-RNAi and WT fruits, transcripts of *PE* (Phan *et al.*, 2007), *PG* (Giovannoni *et al.*, 1989), *CEL2* (Lashbrook *et al.*, 1994), *XYL1*(Buanafina *et al.*, 2015), *XTH5* (Miedes and Lorences, 2009), *TBG4* (Smith *et al.*, 2002), *MAN1* (Meli *et al.*, 2010), *PL* (Uluisik *et al.*, 2016), and *EXP1* (Brummell *et al.*, 1999*b*) were detected in B+4 fruits and quantified relative to expression in WT fruits (Fig. 4A–I). The expression levels of all genes except *PE* were down-regulated; notably, *PG*, *TBG4*, *CEL2*, and *XYL1* were down-regulated by more than 80% (Fig. 4A–D). These results suggest that silencing of *SlFSR* may affect tomato cell wall modiﬁcation.

**Fig. 5.**
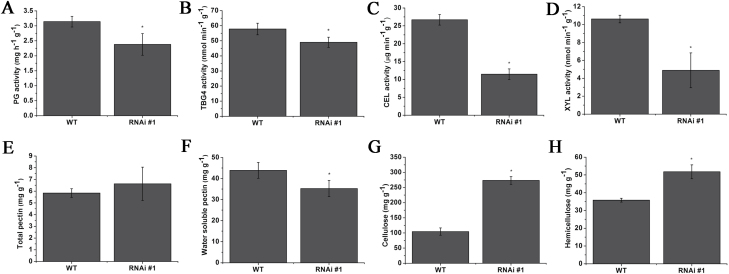
(A–D) Activities of PG, TBG, CEL, and XYL in WT and *SlFSR*-RNAi fruits at the B+4 stage. (E–H) Contents of (E) total pectin, (F) water-soluble pectin, (G) cellulose and (H) hemicellulose in *SlFSR*-RNAi and WT fruits at the B+4 stage. Each value represents the mean ±SE of three replicates. Asterisks indicate significant differences (*P*<0.05) between WT and RNAi lines.

The significant inhibition of the expression of cell wall modiﬁcation-related genes in *SlFSR*-RNAi fruits suggests that the activity of relevant enzymes is also reduced in these lines. Similar to the reduced *PG*, *TBG4*, *CEL2*, and *XYL1* expression that was observed at the mRNA level (see above), reduced activity of PG, TBG, CEL, and XYL at the protein level was observed in *SlFSR*-RNAi fruits, relative to WT fruits, at the B+4 stage (Fig. 5A–D). The total pectin content in *SlFSR*-RNAi fruits at the B+4 stage was not significantly different from that in WT fruits (Fig. 5E). However, water-soluble pectin was lower in *SlFSR*-RNAi fruits (Fig. 5F). Moreover, the cellulose and hemicellulose contents in *SlFSR*-RNAi fruits at the B+4 stage were higher than their respective concentrations in WT fruits (Fig. 5G, H). These results suggest that down-regulation of cell wall modiﬁcation-related genes and changes in the activity of cell wall modiﬁcation-related enzymes and cell wall components in *SlFSR*-RNAi fruits contribute to the prolonged shelf-life observed for RNAi fruits.

### Overexpression of *SlFSR* cannot restore the course of fruit ripening in the *rin* mutant

**Fig. 6.**
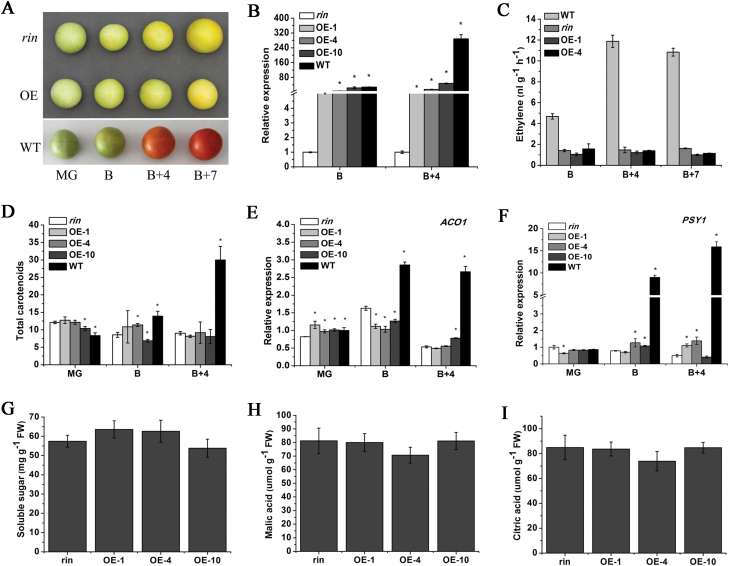
Overexpression of *SlFSR* in *rin* leads to no obvious phenotypic changes. (A) Color of *rin*, *SlFSR*-overexpressing transgenic *rin* (OE), and WT fruits at the MG, B, B+4, and B+7 stages. (B) Relative levels of *SlFSR* mRNA in *rin* (control), WT, and *SlFSR*-overexpressing fruit at the B and B+4 stages. The expression data for *rin* fruits were normalized to a value of 1. (C–F) Ethylene production (C), accumulation of carotenoids (D), expression of *ACO1* (E), and expression of *PSY1* (F) in *rin*, WT and *SlFSR*-overexpressing transgenic *rin* fruits. (G–I) Analysis of flavor compounds in WT, *rin* and *SlFSR*-overexpressing transgenic *rin* fruits. The expression data for *rin* fruits were normalized to a value of 1. Each value represents the mean ±SE of three replicates. Asterisks indicate significant differences (*P*<0.05) between *rin* and the other lines.

To explore the function of *SlFSR* in fruit ripening and color development in more depth, a *SlFSR*-overexpression vector was constructed and transformed into the tomato mutant *rin*. Transgenic *rin* lines (OE-1, OE-4, and OE-10) were generated; *SlFSR* mRNA accumulated to a higher level in these lines than in *rin;* expression in the transgenic lines was similar to that of WT at the B stage (Fig. 6B). However, there was no difference in fruit color between the transgenic *rin* lines and *rin* (Fig. 6A). In addition, the *SlFSR*-overexpressing lines did not exhibit obvious differences in ethylene production from B to B+7 stage, like *rin*; in contrast, WT fruits showed a rapid and considerable increase in ethylene production at the B+4 stage (Fig. 6C). Moreover, overexpression of *SlFSR* resulted in little change in carotenoid accumulation compared with *rin*, whereas a significant increase was observed in WT fruits at the B and B+4 stages (Fig. 6D). Similarly, overexpression of *SlFSR* did not activate the expression of *ACO1* and *PSY1*, while a dramatic increase in transcripts of *ACO1* and *PSY1* was observed in WT fruits at the B and B+4 stages (Fig. 6E, F). Nevertheless, some flavor compounds, such as sugar, malic acid, and citric acid, showed no significant change in *SlFSR*-overexpressing transgenic lines compared with *rin* (Fig. 6G–I).These results indicate that the course of fruit ripening of the mutant *rin* cannot be restored to a WT-like phenotype by the overexpression of *SlFSR*.

### Overexpression of *SlFSR* shortens the shelf-life of transgenic *rin* fruits

**Fig. 7.**
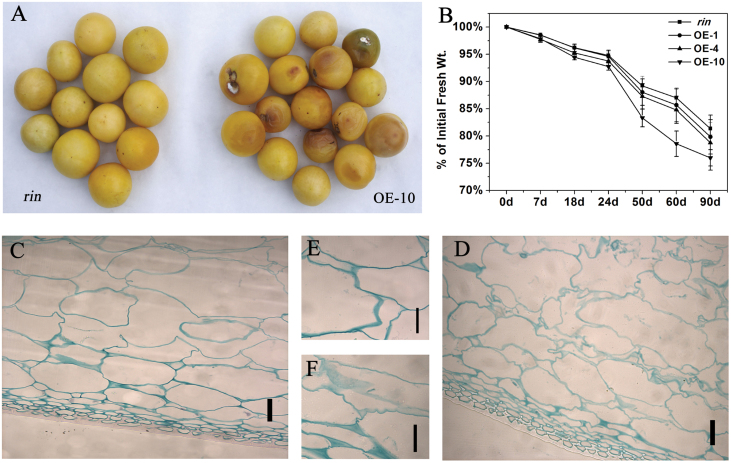
Overexpression of *SlFSR* alters tomato fruit cell wall composition and shortens the shelf-life. (A) Appearance of *rin* and *SlFSR*-overexpression transgenic *rin* fruits harvested at B+4 and stored at room temperature for 90 days. (B) Fresh weight loss of *rin* and *SlFSR*-overexpressing fruits during storage. (C, E) Microscopic observations of *rin* tomato fruit stored for 60 days. (D, F) Microscopic observations of *SlFSR*-overexpressing tomato fruit stored for 60 days. (C, D) Bar=50 μm; (E, F) Bar=25 μm.

To investigate the effects of *SlFSR* overexpression on fruit shelf-life, fruits of the mutant *rin* and *SlFSR*-overexpressing transgenic *rin* were harvested at the breaker stage and stored at room temperature. Despite the lack of discernible differences in ripening and color development between *SlFSR*-overexpressing lines and *rin* fruits (see (Fig. 6A), the overexpressing lines exhibited a shorter fruit shelf-life than *rin*. *SlFSR*-overexpressing lines showed visible signs of rot and deterioration 3 months after harvest; by contrast, no obvious signs of deterioration or rot were observed in *rin* fruit stored under the same conditions for 3 months (Fig. 7A). The fresh weight of the fruits was also measured during post-harvest storage. *SlFSR*-overexpressing lines showed a significantly larger decrease in fruit fresh weight than *rin* (Fig. 7B). Microscopic examination of pericarp samples taken after 90 days of post-harvest storage revealed ruptured cells with irregular shape in the *SlFSR*-overexpressing lines (Fig. 7C), while in *rin* the cells were comparatively round shaped and normal in appearance (Fig. 7D). In addition, the cell walls of the fruit of *SlFSR*-overexpressing lines were degraded, whereas *rin* fruit showed a normal cell wall structure (Fig. 7E, F). These results suggest that the overexpression of *SlFSR* in *rin* significantly shortens fruit shelf-life.

### Expression profiles of cell wall modiﬁcation-related genes in *SlFSR*-overexpressing fruits

**Fig. 8.**
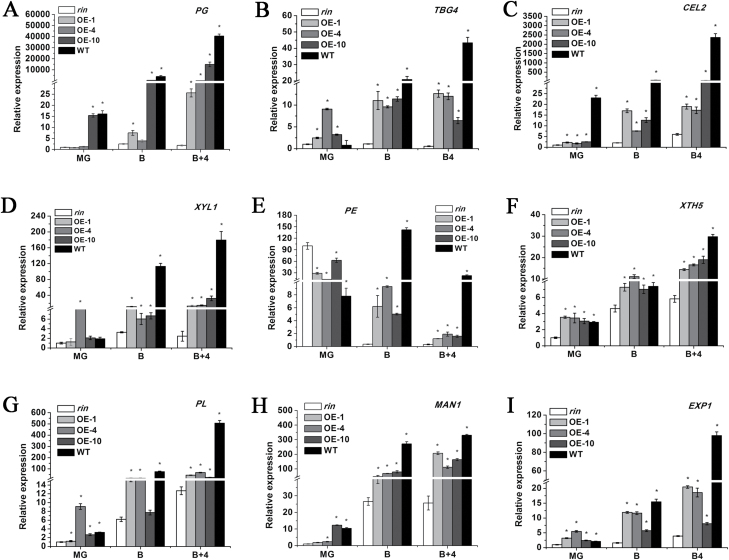
(A–I) Relative expression profiles of cell wall metabolism genes in the pericarp of *rin*, WT, and *SlFSR*-overexpressing transgenic *rin* tomato fruits. The expression data for *rin* fruits were normalized to a value of 1. Each value represents the mean ±SE of three replicates. Asterisks indicate significant differences (*P*<0.05) between *rin* and the other lines.

**Fig. 9.**
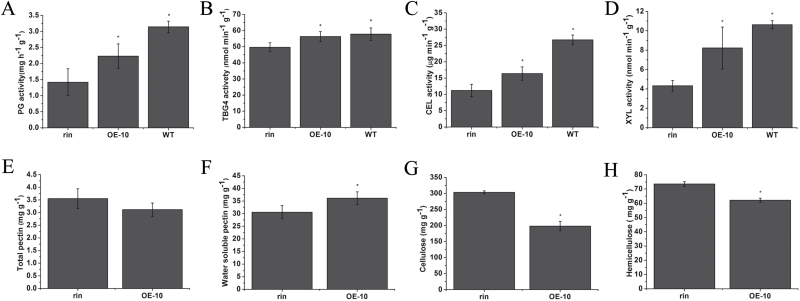
(A–D) Activities of PG, TBG, CEL, and XYL in the pericarp of *rin* and *SlFSR*-overexpressing transgenic *rin* tomato fruits at the B+4 stage. (E–H) Contents of (E) total pectin, (F) water-soluble pectin, (G) cellulose, and (H) hemicellulose in *SlFSR*-overexpressing and *rin* fruits at the B+4 stage. Each value represents the mean ±SE of three replicates. Asterisks indicate significant differences (*P*<0.05) between *rin* and *SlFSR*-overexpressing lines.

In order to further ascertain the molecular mechanisms of the shortened fruit shelf-life in *SlFSR*-overexpressing tomato lines, the expression profiles of cell wall modiﬁcation-related genes, including *PG*, *PE*, *TBG4*, *CEL2*, *XYL1, XTH5*, *EXP1*, MAN1, and *PL*, were examined in *SlFSR*-overexpressing, *rin*, and WT fruits (Fig. 8A–I). The transcript levels of all these genes were up-regulated in *SlFSR*-overexpressing transgenic *rin* fruits compared with *rin* fruits at the B and B+4 stages, but did not reach the levels observed in WT fruits. The activity of the protein products of some of these genes was assessed: PG, TBG, CEL, and XYL showed a significant increase in activity in WT and *SlFSR*-overexpressing fruits, relative to the activity in *rin* fruits, at the B+4 stage (Fig. 9A–D). The total pectin content in *SlFSR*-overexpressing fruits at the B+4 stage was slightly (although not significantly) lower than that in *rin* (Fig. 9E). By contrast, the water-soluble pectin content was higher in *SlFSR*-overexpressing fruit than in *rin* (Fig. 9F), suggesting that the down-regulation of *SlFSR* promotes pectin degradation. Moreover, the cellulose and hemicellulose contents in *SlFSR*-overexpressing fruits at the B+4 stage were significantly lower than in *rin* (Fig. 9G, H). These results indicate that the expression of cell wall modiﬁcation-related genes is positively regulated by *SlFSR*, and that the overexpression of *SlFSR* in *rin* indeed accelerates the degradation of the fruit cell wall.

## Discussion

Tomato fruits are rich in vitamins, ﬁber, minerals, and antioxidants, which are key components of human nutrition. In tomato production, great losses often occur as a result of over-softening and subsequent fungal infections during post-harvest transportation and storage, and post-harvest loss is one of the major problems in tomato production. To date, numerous genes have been reported to control tomato fruit growth, ripening ,and softening (Brummell and Harpster, 2001; Karlova *et al.*, 2014).

Plant-specific GRAS proteins play critical and diverse roles in growth and development (Sun *et al.*, 2012). Here, we report on a new GRAS gene, designated as *SlFSR* (*fruit shelf-life regulator*), whose mRNA specifically accumulates in ripening fruits, implying its potential role in tomato fruit ripening and/or softening (Fig. 1). Interestingly, silencing *SlFSR* in tomato greatly prolonged the shelf-life and reduced cell degradation of fruits, as confirmed by the decreased expression of multiple cell wall modification-related genes and reduced PG, TBG, CEL, and XYL activities, but did not exert a significant influence on the normal fruit ripening phenotype (Figs 2–5). In addition, transgenic *SlFSR*-overexpressing lines exhibited a similar inhibited ripening process to that of the *rin* mutant and had comparable levels of ethylene and carotenoids production to *rin* (Fig. 6), suggesting that the overexpression of *SlFSR* is unable to recover the impaired ripening phenotype of *rin*. Essentially, overexpression of *SlFSR* in the *rin* background significantly reduced the shelf-life and increased the rate of water loss in stored fruits by up-regulating the expression of multiple cell wall modification-related genes (Figs 6–8); increased activities of PG, TBG, CEL, and XYL were detected in *SlFSR*-overexpressing fruits (Fig. 9).

It has been well documented that cell wall modiﬁcation-related proteins, including PG, TBG4, CEL2, XYL1, PE, XTH5, PL, MAN1, and EXP1, function in cell wall disruption, and are generally considered as key factors in the changes that occur to the primary cell wall during fruit ripening (Owino *et al.*, 2005; Brummell, 2006; Vicente *et al.*, 2007). For instance, PG is involved in polyuronide solubilization and depolymerization during ripening, but is not necessary or sufficient for tomato fruit ripening. However, resistance to post-harvest pathogens cracking and shelf-life were improved in PG-RNAi lines (Kramer *et al.*, 1992; Hadfield and Bennett, 1998). CELs are involved in fruit ripening, tissue abscission, cell extension and differentiation (Flors *et al.*, 2007). Greatly increased levels of *CEL2* mRNA were found at the onset of ripening, but the suppression of *CEL2* did not affect the changes in fruit softening (Brummell *et al.*, 1999*a*). Subsequently, Flors *et al.* (2007) found that lack of both *CEL1* and *CEL2* decreases susceptibility to *Botrytis cinerea* infection in tomato. XYL is involved in cell wall degradation, via participation in the breakdown of xylans (Buanafina *et al.*, 2015). XTHs are believed to be related to the maintenance of the structural integrity of the cell wall, and *XTH5* is evidently related to fruit ripening (Miedes and Lorences, 2009). PE is involved in pectin depolymerization and affects tissue integrity in over-ripe fruit (Brummell and Harpster, 2001). Silencing *PL* at the mRNA level improved tomato shelf-life; this improvement was caused by differences in the levels of total pectin and soluble pectin (Uluisik *et al.*, 2016). MAN is involved in fruit shelf-life, without any negative effect on vegetative growth, fruit development, days to maturity, seed production, and yield (Meli *et al.*, 2010). β-Galactosidase, encoded by *TBG4*, plays a role in the hydrolysis of galactan side-chains of pectic polysaccharides. Down-regulation of *TBG4* results in significantly greater fruit firmness compared with WT fruits (Smith *et al.*, 2002). The suppression and overexpression of *EXP1* mRNA and protein accumulation caused changes in fruit softening during ripening, and multiple changes in cell wall polysaccharide metabolism (Brummell *et al.*, 1999*b*). Therefore, given that these cell wall modiﬁcation-related genes/proteins play important roles in fruit softening, shelf-life, and resistance to post-harvest pathogens, their reduced expression/activities may extend the shelf-life of *SlFSR*-RNAi fruits. In contrast, their increased expression/activities may result in the shortened shelf-life of *SlFSR*-overexpressing fruits. Taken together, these results suggest that the SlFSR transcription factor may participate in the modulation of tomato cell wall metabolism and may affect fruit shelf-life by regulating the expression of genes related to cell wall modification.

Although changes in the expression level of *SlFSR* could significantly influence the shelf-life of tomato fruits, the ripening phenotype of the *SlFSR*-RNAi lines and *SlFSR*-overexpressing transgenic *rin* fruits showed no significant changes. Generally, ethylene plays a critical role during fruit ripening and softening in climacteric fruits (Hiwasa *et al.*, 2003; Ergun *et al.*, 2005; Nishiyama *et al.*, 2007); however, it is not the only key regulator of fruit ripening. RIN is believed to also act as a key ripening regulator by acting upstream of both ethylene-dependent and ethylene-independent pathways (Vrebalov *et al.*, 2002). The *rin* mutation has been investigated extensively in studies to identify genes associated with the ripening process. RIN-targeted genes participate in a range of fruit ripening-associated metabolic and regulatory mechanisms, including cell wall metabolism and ethylene signaling, suggesting that RIN controls fruit ethylene production and softening through the transcriptional regulation of ethylene biosynthesis genes and cell wall-modifying genes during ripening (Fujisawa *et al.*, 2011). To date, 342 genes positively regulated by RIN and 473 genes negatively regulated by RIN have been identified (Fujisawa *et al.*, 2012). Moreover, 241 genes that are direct targets of RIN have been identiﬁed (Fujisawa *et al.*, 2013). Most of the positively regulated genes contained possible RIN-binding (CArG-box motif) sequences [C(C/T)(A/T)6(A/G)G] in their promoters (Ito *et al.*, 2008). Subsequently, a potential binding site for RIN was found in the promoter region of the *SlFSR* gene. The *SlFSR* gene promoter has three typical CArG-box sequences [C(A/T)8G] and one intermediate CArG-box sequence [CC(A/T)6AG] (Supplementary Fig S1), suggesting that that *SlFSR* expression can be regulated by RIN. Moreover, the expression level of *SlFSR* was down-regulated in the *rin* mutant compared with WT. Therefore, RIN may directly regulate the expression of *SlFSR*, providing an important clue to elucidate the complicated transcriptional cascade for tomato cell wall modiﬁcation.

**Fig. 10.**
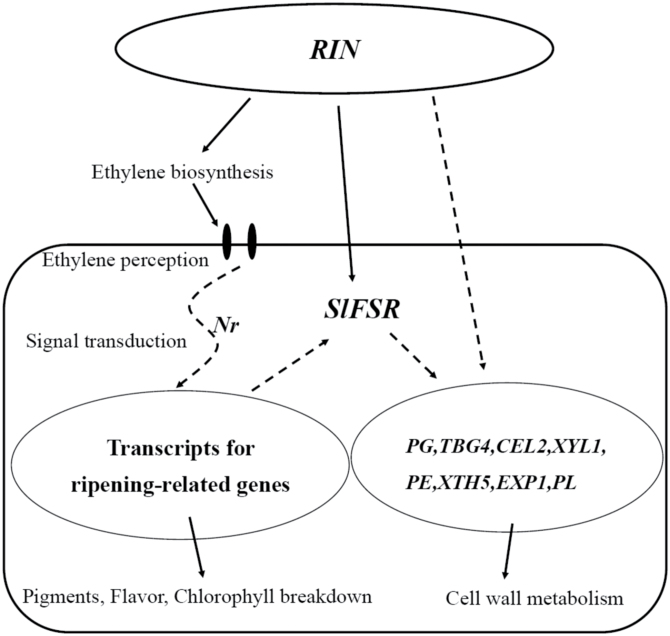
Proposed model depicting the regulation of the *SlFSR* gene and its function in tomato fruit shelf-life.

In conclusion, we have identiﬁed an important fruit shelf-life regulator, *SlFSR*. The results obtained from our experiments with both *SlFSR*-RNAi and *SlFSR*-overexpressing fruits enable us to conclude that there is a link between *SlFSR* and fruit shelf-life in tomato. We have attempted to summarize our results in a model to explain the potential role of *SlFSR* in regulating tomato fruit cell wall metabolism (Fig. 10). In brief, our results provide a valuable opportunity to deepen understanding of the genetic mechanism underlying this significant agronomic trait and to facilitate molecular breeding in tomato. Recognition of the role of *SlFSR* in post-harvest storage may be conducive to the design and development of approaches to limit losses during fruit storage, handling. and delivery.

## Supplementary data

Supplementary data are available at *JXB* online.

Table S1. Specific primer sequences used in this study.

Fig. S1. Putative *cis*-elements enriched in the promoter of the *SlFSR* gene.

Supplementary Table S1

Supplementary Figure S1
