# Supplementary material for: The SlFSR gene controls fruit shelf-life in tomato
Source: J Exp Bot. 2018 Apr 4;69(12):2897–909. doi: 10.1093/jxb/ery116 (PMC5972576; doi:10.1093/jxb/ery116)
Supplement: Supplementary Table S1 [file ery116_suppl_supplementary_table_s1.pdf]

Running title: *SlFSR* plays a role in tomato fruit shelf-life

Title:

The *SlFSR* gene controls fruit shelf-life in tomato

Authors: Lincheng Zhang*, Mingku Zhu*, Lijun Ren*, Anzhou Li, Guoping Chen and Zongli Hu

Key Laboratory of Biorheological Science and Technology (Chongqing University), Ministry of Education, Bioengineering College, Chongqing University, Chongqing 400044, People's Republic of China

*These authors contributed equally to this work

Guoping Chen(Author for Correspondence**)：** Room 523, Bioengineering College, Chongqing University, Campus B, 174 Shapingba Main Street, Chongqing 400030, P.R. China; **Tel:** 00862365112674; **Fax:** 00862365112674; **E-mail:** Guoping Chen: chenguoping@cqu.edu.cn

Zongli Hu (Author for Correspondence**):** Room 523, Bioengineering College, Chongqing University, Campus B, 174 Shapingba Main Street, Chongqing 400030, P.R. China; **Tel:** 00862365112674; **Fax:** 00862365112674; **E-mail:** Zongli Hu: huzongli71@163.com

The e-mail addresses of the authors:

Lincheng Zhang: zhanglc201312@163.com

Mingku Zhu: mingkuzhu007@126.com

Lijun Ren: renlijun403@163.com

Anzhou Li: lianzhou6375@163.com

Received 20 November 201

Table S1 Specific primer sequences used for gene amplification and qRT-PCR analysis.

Primer code	Primer sequences (5' →3')	Application
CAC-Q-F	CCTCCGTTGTGATGTAACTGG	Internal standard gene for Quantitative
CAC-Q-R	ATTGGTGGAAAGTAACATCATCG	RT-PCR in tomato development
EF1α-Q-F	TACTGGTGGTTTTGAAGCTG	Internal standard gene for Quantitative
EF1α-Q-R	AACTTCCTTCACGATTTCATCATA	RT-PCR under ACC treatment
SlFSR-Q-F	GAACTCTACAACCATTCCCCTCC	Quantitative RT-PCR analysis for
SlFSR-Q-R	TAATAGTTTCTCCAAACATGCTCCA	*SlFSR*
SlFSR-RNAi-F	CGGGGTACCATCGATATTGGGAGCCTTGATGGA	To establish *SlFSR* RNAi lines; added *Kpn* I and *Cla* I site underlined
SlFSR-RNAi-R	CCGCTCGAGTCTAGACGCGAGACGAAAGTGCA	To establish *SlFSR* RNAi lines; added *Xho* I and *Xba* I site underlined
SlFSR-over-F	GCTCTAGACTCTTCACCCGCAGACCTAA	To establish *SlFSR* overexpression

		lines; added *Xba* I site underlined
SlFSR-over-R	CGAGCTCCAATGGACCACTTTGAGAATGA	To establish *SlFSR* overexpression lines; added *Sac I* site underlined
NPT II-F	GACAATCGGCTGCTCTGA	Positive transgenic plants detection
NPT II-R	AACTCCAGCATGAGATCC	
PSY1-Q-F	AGAGGTGGTGGAAAGCAA	Quantitative RT-PCR analysis for
PSY1-Q-R	TCTCGGGAGTCATTAGCAT	ethylene- and ripening-related genes
ACO1-Q-F	ACAAACAGACGGGACACGAA	
ACO1-Q-R	CTCTTTGGCTTGAAACTTGA	
E8-Q-F	GGCACCATTCAACATACCG	
E8-Q-R	CTTTCACCGAAGAAGCACG	
RIN-Q-F	GGAACCCAAACTTCATCAGA	
RIN-Q-R	TTGTCCCAAATCCTCACCTA	
LOXB-Q-F	TGCTACAATGACTTGGGTGAA	
LOXB-Q-R	CCTGTCCTGCCTCTACG	
PG-Q-F	ATACAACAGTTTTCAGCAGTTCAAGT	Quantitative RT-PCR analysis for
PG-QR	GGTTTTCCACTTTCCCCTACTAA	cell wall metabolism-related genes
PE1-Q-F	GCTTGCGTCTTTGACAACTCAGG	
PE1-Q-R	GTGCCACCACTGCATTCGCTAT	
XYL1-Q-F	TGATCGGCAATTATGAAGGTATTC	
XYL1-Q-R	CAGCACATCCTGGCTTGTAAAT	
XTH5-Q-F	CCACCACCAGAGTGCGAGAT	
XTH5-Q-R	TTTTCTTAGGATGACGATGTCCG	
CEL2-Q-F	ACACATTGCCAAACGTCAGGT	
CEL2-Q-R	CCCCTATGGTGAATCCTTTGTG	
EXP1-Q-F	CCCTCCTCGCCCTCACTTT	
EXP1-Q-R	TCTGATTCCTCCTTGCTTTCG	
PL-Q-F	GCGATCAGGAGTTAGAACTGG	
PL-Q-R	AATCCCCTTTTGCTTTGGTT	
TBG4-Q-F	AAATGGTGAAGGCGTAGGTCG	
TBG4-Q-R	AGGTTGTCCGCAGTTAGTCTGG	
MAN1-Q-F	ACACCGTCCTCCTGAGATTGG	
MAN1-Q-R	GAGCCTCTGCTTTCCACTTTAATC	
