# Supplementary material for: The SlFSR gene controls fruit shelf-life in tomato
Source: J Exp Bot. 2018 Apr 4;69(12):2897–909. doi: 10.1093/jxb/ery116 (PMC5972576; doi:10.1093/jxb/ery116)
Supplement: Supplementary Figure S1 [file ery116_suppl_supplementary_figure_s1.pdf]

Running title: *SlFSR* plays a role in tomato fruit shelf-life

Title:

The *SlFSR* gene controls fruit shelf-life in tomato

Authors: Lincheng Zhang*, Mingku Zhu*, Lijun Ren*, Anzhou Li, Guoping Chen and Zongli Hu

Key Laboratory of Biorheological Science and Technology (Chongqing University), Ministry of Education, Bioengineering College, Chongqing University, Chongqing 400044, People's Republic of China

---

*These authors contributed equally to this work

Guoping Chen(Author for Correspondence)： Room 523, Bioengineering College, Chongqing University, Campus B, 174 Shapingba Main Street, Chongqing 400030, P.R. China; **Tel:** 00862365112674; **Fax:** 00862365112674; **E-mail:** Guoping Chen: chenguoping@cqu.edu.cn

Zongli Hu (Author for Correspondence)**:** Room 523, Bioengineering College, Chongqing University, Campus B, 174 Shapingba Main Street, Chongqing 400030, P.R. China; **Tel:** 00862365112674; **Fax:** 00862365112674; **E-mail:** Zongli Hu: huzongli71@163.com

The e-mail addresses of the authors:

Lincheng Zhang: zhanglc201312@163.com

Mingku Zhu: mingkuzhu007@126.com

Lijun Ren: renlijun403@163.com

Anzhou Li: lianzhou6375@163.com

Received 20 November 201

Figure legends

Fig. S1 Putative cis-elements enriched in the promoter of *SlFSR* gene. The start codon is shown as ATG

Fig. S1

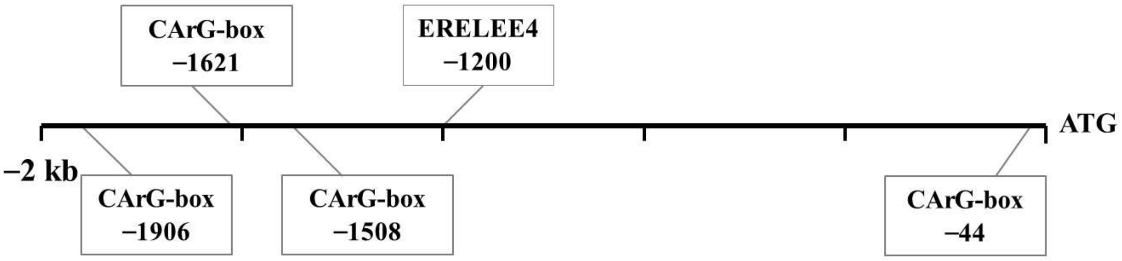

CArG-box [C(C/T)(A/T)6(A/G)G]: binding site for RIN transcription factor.
–1906, CATTTTTAAG; –1621, CCATTTTAAG; –1508, CAATATTTTG, –44, CATTTTATTG.
ERELEE4 (AWTTCAAA): ethylene responsive element. –1200, AATTCAAA. W=A/T.
